# How many meals and snacks do Brazilians eat a day? Findings from the 2017-2018 *Brazilian National Dietary Survey*

**DOI:** 10.1590/0102-311XEN009923

**Published:** 2024-02-19

**Authors:** Paulo Rogério Melo Rodrigues, Luana Silva Monteiro, Thaís Meirelles de Vasconcelos, Luiz Eduardo da Silva Gomes, Iuna Arruda Alves, Valéria Troncoso Baltar, Bartira Mendes Gorgulho, Edna Massae Yokoo, Rosely Sichieri, Rosangela Alves Pereira

**Affiliations:** 1 Faculdade de Nutrição, Universidade Federal de Mato Grosso, Cuiabá, Brasil.; 2 Universidade Federal do Rio de Janeiro, Macaé, Brasil.; 3 Programa de Pós-graduação em Saúde Coletiva, Universidade Estadual do Ceará, Fortaleza, Brasil.; 4 Instituto de Matemática, Universidade Federal do Rio de Janeiro, Rio de Janeiro, Brasil.; 5 Instituto de Nutrição Josué de Castro, Universidade Federal do Rio de Janeiro, Rio de Janeiro, Brasil.; 6 Instituto de Saúde Coletiva, Universidade Federal Fluminense, Niterói, Brasil.; 7 Instituto de Medicina Social, Universidade do Estado do Rio de Janeiro, Rio de Janeiro, Brasil.

**Keywords:** Meals, Food Consumption, Food Habits, Nutrition Surveys, Refeições, Consumo Alimentar, Hábitos Alimentares, Inquéritos Nutricionais, Comidas, Consumo Alimentario, Conducta Alimentaria, Encuestas Nutricionales

## Abstract

The habit of eating specific meals has been addressed in several studies, but the evaluation of meal patterns has received less attention. This study aimed to describe the meal patterns of the Brazilian population. A complex sampling design was used to select the 46,164 ≥ 10-year-old individuals examined in the *Brazilian National Dietary Survey*. Food consumption was assessed by two non-consecutive 24-hour recalls applied throughout a one-week period. The exploratory data analysis approach was used to determine the meal patterns, i.e., how individuals combined the main meals (breakfast, lunch, dinner) and snacks (morning, afternoon, evening/night) throughout the day. The most common meal patterns were three main meals + one snack, reported by 25.1% of the individuals, and three main meals + two snacks (24.6%). Other meal patterns identified were: three main meals + three snacks (18.5%); three main meals and no snacks (10.9%); one or two main meals + two snacks (7.4%); one or two main meals + one snack (6.9%); one or two main meals + three snacks (4.2%); and one or two main meals and no snacks (2.3%). Meal patterns varied according to gender and age group, and on typical versus atypical food consumption days. We found that eight patterns characterized the daily meal consumption in Brazil. Furthermore, around 80% of the population had three main meals every day and about 13% did not report having any snacks. The characterization of meal habits is important for tailoring and targeting health promotion actions.

## Abstract (pt)

Os hábitos de consumo de refeições específicas têm sido abordados em diversos estudos, no entanto, a avaliação dos padrões refeições tem recebido menos atenção. O objetivo deste estudo foi descrever os padrões de refeições da população brasileira. Um desenho amostral complexo foi utilizado para selecionar os 46.164 indivíduos de ≥ 10 anos examinados no *Inquérito Nacional de Alimentação* de 2017-2018. O consumo alimentar foi avaliado por meio de dois recordatórios de 24 horas não consecutivos, aplicados durante um período de uma semana. A análise exploratória de dados foi utilizada para determinar os padrões de refeições, ou seja, como os indivíduos combinam as principais refeições (café da manhã, almoço, jantar) e lanches (manhã, tarde, noite) ao longo do dia. Os padrões de refeições mais frequentes foram três refeições principais + um lanche, conforme relatado por 25,1% dos indivíduos, e três refeições principais + dois lanches (24,6%). Outros padrões de refeições identificados foram: três refeições principais + três lanches (18,5%); três refeições principais e nenhum lanche (10,9%); uma ou duas refeições principais + dois lanches (7,4%); uma ou duas refeições principais + um lanche (6,9%); uma ou duas refeições principais + três lanches (4,2%); e uma ou duas refeições principais e nenhum lanche (2,3%). Os padrões de refeições variaram de acordo com o sexo e a faixa etária, e nos dias típicos em comparação com os atípicos de consumo alimentar. Verificou-se que oito padrões caracterizaram o consumo diário de refeições no Brasil. Além disso, cerca de 80% da população realizava três refeições principais diárias e cerca de 13% reportaram não lanchar. A caracterização dos padrões de refeições é importante para adequar e direcionar ações de promoção da saúde.

## Abstract (es)

Los hábitos alimenticios específicos se han abordado en varios estudios, sin embargo, poco se sabe sobre la evaluación de los patrones de alimentación. El objetivo de este estudio fue describir el patrón de alimentación de la población brasileña. Se utilizó un diseño de muestra complejo para seleccionar a 46.164 individuos de ≥ 10 años quienes participaron en la *Encuesta Nacional de Alimentación* 2017-2018. El consumo alimentario se evaluó mediante dos registros de 24 horas no consecutivos, aplicados durante una semana. Para determinar el patrón de alimentación, se aplicó el análisis exploratorio, es decir, cómo las personas combinan las comidas principales (desayuno, almuerzo, cena) y las meriendas (mañana, tarde, noche) a lo largo del día. Los patrones de alimentación más frecuentes fueron tres comidas principales + una merienda según informan el 25,1% de los individuos, y tres comidas principales + dos meriendas (24,6%). Otros patrones identificados destacaron tres comidas principales + tres meriendas (18,5%); tres comidas principales sin merienda (10,9%); una o dos comidas principales + dos meriendas (7,4%); una o dos comidas principales + una merienda (6,9%); una o dos comidas principales + tres meriendas (4,2%); y una o dos comidas principales sin merienda (2,3%). Los patrones de alimentación tuvieron una variación según el sexo y el grupo de edad, y en días típicos en comparación con los atípicos de consumo de alimentos. Se encontró que ocho patrones caracterizan el consumo diario de comidas en Brasil. Por lo tanto, aproximadamente el 80% de la población tienen tres comidas principales al día y aproximadamente el 13% informan que no tienen merienda. Es importante caracterizar los patrones de alimentación para adaptar y orientar las acciones de promoción de la salud.

## Introduction

The study of eating occasions aims to describe some of the various aspects related to food or beverage consumption. Generally, eating occasions are categorized according to the amount and type of food consumed and to the timing of consumption ^1^. Daily food consumption is usually characterized by three main meals - namely breakfast, lunch, and dinner - that can be interspersed with snacks ^2^ ^,^ ^3^.

The interest in this topic is related to the role possibly played by the number and timing of meals on metabolism ^4^ ^,^ ^5^, weight gain ^6^ ^,^ ^7^, and insulin-related metabolic disorders ^8^ ^,^ ^9^. In addition, the habit of skipping main meals and replacing them with snacks has been associated with a low-quality diet ^8^ ^,^ ^9^, body adiposity ^1^, cardiovascular risk ^3^, noncommunicable chronic diseases ^10^, and sleep disorders ^11^. The daily frequency of eating occasions has been inversely associated with concentrations of total cholesterol and low-density lipoprotein cholesterol ^4^ and C-reactive protein ^5^. Additionally, a 10-year follow-up study showed that breakfast consumption may help prevent weight gain ^6^. Furthermore, the habit of eating lunch late (i.e., at 4:30 p.m.) was associated with impaired free cortisol concentrations throughout the day and reduced resting energy expenditure, fasting carbohydrate oxidation, glucose tolerance, and in the thermic effect of food ^7^.

Several studies have explored the habits related to specific meals ^3^ ^,^ ^8^, but few have particularly focused on the assessment of meal patterns, i.e., the combination of different eating occasions throughout the day ^12^. In Brazil, local surveys have assessed the meal patterns of adolescents ^13^ ^,^ ^14^ ^,^ ^15^ ^,^ ^16^ ^,^ ^17^. To date, no study with a nationally representative sample has explored the meal and snack patterns of the Brazilian population, which are addressed in this study, based on an analysis of data from the 2017-2018 *Brazilian National Dietary Survey* (INA).

## Methods and materials

### Study design and sample

This study was based on data from the INA included in the 2017-2018 *Brazilian Household Budget Survey* (POF), carried out by the Brazilian Institute of Geography and Statistics (IBGE). The random sampling plan adopted in the POF is based on a master sample composed of a set of census sectors (primary sample units) stratified according to geographical area, household location (urban or rural), and household income. A subsample of households was selected by simple random sampling to form the INA sample, consisting of all residents aged 10 years or over. In the INA 2017-2018, data on food consumption were collected from 46,164 individuals from 20,112 households. The survey was carried out over a 12-month period, so that all geographic and socioeconomic strata were covered in the four quarters, allowing researchers to analyze the coverage of seasonal variation in the events ^18^.

### Ethics statement

This study was deemed exempt by the Research Ethics Committee of the Institute of Social Medicine, State University of Rio de Janeiro (review n. 4,316,087), under the Brazilian National Health Council *Resolution n. 46/2012* and *Operational Act n. 001/2013*, since the data used is de-identified and publicly available (http://www.ibge.gov.br).

### Food consumption assessment

Food consumption was assessed using the 24-hour diet recall (24hR) on two non-consecutive days (84% of participants responded to the second 24hR) chosen over one week. Informers were asked to report all food and beverages (including water) they had consumed the day before the household interview, which was conducted based on the Automated Multiple-Pass Method ^19^, using a computational tool designed specifically for this assessment. For each food and drink, information was requested on the amount consumed, place and time of consumption, and eating occasion (breakfast, lunch, dinner, or snack) ^18^.

In this analysis, “breakfast”, “lunch”, and “dinner” were considered main meals, while “snacks” were classified as “morning snack” (6:00 a.m.-12:00 a.m.), “afternoon snack” (1:00 p.m.-5:00 p.m.) or “evening snack” (6:00 p.m.-5:00 a.m,), based on the time of consumption ^20^.

The consumption of each meal or snack was represented by a yes-no variable. Thus, using the exploratory data analysis (EDA) ^21^, meal and snack patterns were identified by combining the six eating occasions. The pattern identification and the combination algorithm were implemented in the R programing language, version 3.5.3 (http://www.r-project.org).

Firstly, the individuals were classified according to whether they ate one, two or three main meals during the day. Then, the combination of the main meals with the morning, afternoon, and evening snacks resulted in the combinations of one, two, or three main meals with none, one, two, or three snacks. Of the 64 possible combinations, eight included three main meals, 24 included two main meals, 24 only included only one main meal, and eight did not include any main meals. The latter were disregarded, as their frequencies were negligible (0.1%). Box 1 shows the 56 possible combinations considered and used to identify the meal and snack patterns. These combinations were classified into broad categories considering the number of main meals and snacks consumed in one day. Then, the categories including one and two main meals were aggregated. The final eight meal patterns were defined based on their interpretability and the frequency with which they were reported by the population. The same process was applied to data collected during days 1 and 2 of the 24hR, and as no differences were found between the meal patterns identified during these two days (Supplementary Material: https://cadernos.ensp.fiocruz.br/static//arquivo/1678-4464-csp-40-02-EN009923-s.pdf), only data from the first 24hR were reported.

**Box 1:** Possible combinations * of main meals and snacks and identified meal consumption patterns. *Brazilian National Dietary Survey*, 2017-2018.

POSSIBLE COMBINATIONS	COMBINATIONS OF MAIN MEALS AND SNACKS (n = 56)	BROAD CATEGORIES OF MEAL AND SNACK PATTERNS (n = 12)	IDENTIFIED MEAL AND SNACK PATTERNS (n = 8)
Main meals without snacks (n = 7)	Breakfast + lunch + dinner and no snacks	Three main meals and no snacks	Three main meals and no snacks
	Breakfast + lunch and no snacks	Two main meals and no snacks	One or two main meals and no snacks
	Breakfast + dinner and no snacks		
	Lunch + dinner and no snacks		
	Breakfast and no snacks	One main meal and no snacks	
	Lunch and no snacks		
	Dinner and no snacks		
Main meals with one snack (n = 21)	Breakfast + lunch + dinner + morning snack	Three main meals + one snack	Three main meals + one snack
	Breakfast + lunch + dinner + afternoon snack		
	Breakfast + lunch + dinner + evening snack		
	Breakfast + lunch + morning snack	Two main meals + one snack	One or two main meals + one snack
	Breakfast + lunch + afternoon snack		
	Breakfast + lunch + evening snack		
	Breakfast + dinner + morning snack		
	Breakfast + dinner + afternoon snack		
	Breakfast + dinner + evening snack		
	Lunch + dinner + morning snack		
	Lunch + dinner + afternoon snack		
	Lunch + dinner + evening snack		
	Breakfast + morning snack	One main meal + one snack	
	Breakfast + afternoon snack		
	Breakfast + evening snack		
	Lunch + morning snack		
	Lunch + afternoon snack		
	Lunch + evening snack		
	Dinner + morning snack		
	Dinner + afternoon snack		
	Dinner + evening snack		
Main meals with two snacks (n = 21)	Breakfast + lunch + dinner and morning + afternoon snacks	Three main meals + two snacks	Three main meals + two snacks
	Breakfast + lunch + dinner and morning + evening snacks		
	Breakfast + lunch + dinner and afternoon + evening snacks		
	Breakfast + lunch and morning + afternoon snacks	Two main meals + two snacks	One or two main meals + two snacks
	Breakfast + lunch and morning+ evening snacks		
	Breakfast + lunch and afternoon + evening snacks		
	Breakfast + dinner and morning + afternoon snacks		
	Breakfast + dinner and morning + evening snacks		
	Breakfast + dinner and afternoon + evening snacks		
	Lunch + dinner and morning + afternoon snacks		
	Lunch + dinner and morning + evening snacks		
	Lunch + dinner and afternoon + evening snacks		
	Breakfast and morning + afternoon snacks	One main meal + two snacks	
	Breakfast and morning + evening snacks		
	Breakfast and afternoon + evening snacks		
	Lunch and morning + afternoon snacks		
	Lunch and morning + evening snacks		
	Lunch and afternoon + evening snacks		
	Dinner and morning + afternoon snacks		
	Dinner and morning + evening snacks		
	Dinner and afternoon + evening snacks		
Main meals with three snacks (n = 7)	Breakfast + lunch + dinner and morning + afternoon + evening snacks	Three main meals + three snacks	Three main meals + three snacks
	Breakfast + lunch and morning + afternoon + evening snacks	Two main meals + three snacks	One or two main meals + three snacks
	Breakfast + dinner and morning + afternoon + evening snacks		
	Lunch + dinner and morning + afternoon + evening snacks		
	Breakfast and morning + afternoon + evening snacks	One main meal + three snacks	
	Lunch and morning + afternoon + evening snacks		
Dinner and morning + afternoon + evening snacks

* Combinations that did not include any main meals (n = 8) were not considered.

### Statistical analyses

The proportion (and 95% confidence intervals [95%CI]) of individuals with each meal and snack pattern was estimated according to gender, age group (adolescents: 10-19 years old; adults: 20-59 years old; and older adults: ≥ 60 years old), and food consumption day (typical or atypical). This information was provided by the participants at the end of the 24hR. Non-overlapping 95%CI indicated differences in the proportions across the categories of explanatory variables. The analysis considered the sample weights and the effect of the study design using the *Complex Sample* module of the SPSS, version 19 (https://www.ibm.com/).

## Results

In total, 52.1% of the study participants were women, 17.8% were adolescents, 64.6% were adults, and 17.6% were older adults. A total of 88.8% of the study population reported having a typical day of food consumption (Tables 1 and 2).

In total, eight meal and snack patterns were identified: (1) three main meals + one snack, reported by 25.1% of the individuals; (2) three main meals + two snacks (24.6%); (3) three main meals + three snacks (18.5%); (4) three main meals and no snacks (10.9%); (5) one or two main meals + two snacks (7.4%); (6) one or two main meals + one snack (6.9%); (7) one or two main meals + three snacks (4.2%); and (8) one or two main meals and no snacks (2.3%). Overall, 79.1% of the population reported having all three meals, with or without snacks, and 86.7% of participants were classified in patterns including at least one snack (Table 1).

**Table 1:** Distribution (%) of the population according to meal and snack patterns by typical or atypical days of food consumption. *Brazilian National Dietary Survey*, 2017-2018 (n = 46,164).

Meal and snack patterns	Total % (95%CI)	Day of food consumption	
		Typical * % (95%CI)	Atypical ** % (95%CI)
Three main meals *** + one snack	25.1 (24.3; 25.9)	25.8 (25.0; 26.7)	19.4 (17.3; 21.8)
Three main meals + two snacks	24.6 (23.9; 25.4)	25.2 (24.4; 26.0)	19.7 (17.7; 21.9)
Three main meals + three snacks	18.5 (17.7; 19.3)	19.2 (18.4; 20.1)	12.3 (10.8; 14.0)
Three main meals and no snacks	10.9 (10.3; 11.7)	11.1 (10.4; 11.9)	9.4 (7.8; 11.3)
All patterns with the three main meals	79.1 (78.3; 79.9)	81.3 (80.7; 82.1)	60.8 (57.5; 64.1)
One or two main meals + one snack	6.9 (6.5; 7.4)	6.4 (5.9; 6.8)	11.3 (9.8; 13.0)
One or two main meals + two snacks	7.4 (6.9; 7.8)	6.5 (6.1; 6.9)	14.5 (12.8; 16.4)
One or two main meals + three snacks	4.2 (3.9; 4.5)	3.8 (3.5; 4.1)	7.3 (6.2; 8.5)
One or two main meals and no snacks	2.3 (1.8; 2.8)	1.9 (1.6; 2.2)	5.2 (2.7; 9.8)
All patterns with one or two main meals	20.7 (20.0; 21.6)	18.6 (17.8; 19.3)	38.3 (35.1; 41.7)
All patterns with at least one snack	86.7 (85.8; 87.5)	86.9 (86.1; 87.7)	84.6 (80.5; 87.9)

95%CI: 95% confidence interval.

* Typical day of food consumption reported by 88.8% of the population;

** Atypical day of food consumption reported by 11.2% of the population;

*** Main meals: breakfast, lunch, and dinner.

The comparison of the distribution of meal and snack patterns according to typical and atypical food consumption days showed that the patterns that included the three main meals were more frequent on typical days (81.3%) than on atypical days (60.8%). The opposite was observed for all the patterns that included one or two main meals, which were more frequent on atypical days (38.3%) than on typical days (18.6%). The most important differences between typical and atypical food consumption days were found for the patterns “one or two main meals + two snacks” (6.5 *vs.* 14.5%), “three main meals + three snacks” (19.2% vs. 12.3%), and “three main meals + one snack” (25.8% vs. 19.4%) (Table 1).

Patterns including three main meals were more frequent among men (80.5%) than among women (77.9%) and less reported by adolescents (75.1%) than by adults (79.8%) and older adults (80.9%). The meal and snack pattern most frequently reported by men, adolescents, and adults was “three meals + one snack”, and the most frequent pattern among women and older adults was “three meals + two snacks”. A meal consumption pattern including one or two main meals was reported by 24.9% of adolescents, a proportion greater than that found for adults (20.2%) and older adults (19%) (Table 2).

**Table 2:** Distribution (%) of the population according to meal and snack patterns by sex and age group. *Brazilian National Dietary Survey*, 2017-2018 (n = 46,164).

	Men % (95%CI)	Women % (95%CI)	Adolescents % (95%CI)	Adults % (95%CI)	Older adults % (95%CI)
Distribution in the population (%)	47.9	52.1	17.8	64.6	17.6
Meal and snack patterns					
Three main meals * + one snack	26.2 (25.2; 27.2)	24.1 (23.1; 25.1)	25.0 (23.4; 26.7)	25.2 (24.3; 26.2)	24.8 (23.2; 26.4)
Three main meals + two snacks	24.1 (23.1; 25.1)	25.1 (24.2; 26.0)	23.8 (22.4; 25.3)	24.4 (23.5; 25.3)	26.3 (24.8; 27.8)
Three main meals + three snacks	17.9 (17.0; 18.8)	19.0 (18.1; 19.9)	17.2 (16.0; 18.6)	18.5 (17.6; 19.4)	19.7 (18.3; 21.3)
Three main meals and no snacks	12.4 (11.5; 13.3)	9.6 (8.9;10.4)	9.0 (7.8; 10.3)	11.7 (10.9; 12.6)	10.1 (9.0; 11.4)
All patterns with three main meals	80.6 (79.5; 81.5)	77.8 (76.9; 78.8)	75.0 (73.4; 76.7)	79.8 (78.8; 80.6)	80.9 (79.4; 82.4)
One or two main meals + one snack	6.7 (6.1; 7.3)	7.1 (6.6; 7.8)	8.3 (7.3; 9.4)	6.7 (6.1; 7.2)	6.4 (5.5; 7.4)
One or two main meals + two snacks	6.6 (6.1; 7.2)	8.1 (7.5; 8.7)	9.2 (8.2; 10.3)	7.1 (6.5; 7.6)	6.7 (5.8; 7.7)
One or two main meals + three snacks	3.6 (3.2; 4.0)	4.8 (4.4; 5.2)	4.6 (4.0; 5.2)	4.0 (3.6; 4.4)	4.7 (4.0; 5.4)
One or two main meals and no snacks	2.5 (2.0; 3.0)	2.1 (1.6; 2.6)	2.8 (1.8; 4.2)	2.4 (2.0; 2.9)	1.2 (0.9; 1.7)
All patterns with one or two main meals	19.4 (18.4; 20.3)	22.1 (21.1; 23.0)	24.9 (23.2; 26.5)	20.2 (19.2; 21.0)	19.0 (17.6; 20.5)
All patterns with at least one snack	85.0 (84.0; 86.0)	88.2 (87.3; 89.1)	88.1 (86.4; 89.7)	85.7 (84.8; 86.7)	88.6 (87.3; 89.8)

95%CI: 95% confidence interval.

* Main meals: breakfast, lunch, and dinner.

The greatest differences between men and women were found for the set of meal and snack patterns including at least one snack. These patterns were more frequently reported by women (88.2%) than by men (85%). The “three main meals” pattern and the remaining patterns including three main meals were both more frequent among men than women (12.4% vs. 9.6% and 80.6% vs. 77.8%, respectively) (Table 1).

The most important differences between age groups were observed for the set of patterns including one or two main meals, which were more frequently reported by adolescents (24.9%) than by adults (20.2%) and older adults (19%). On the other hand, the patterns including three main meals were more commonly reported by older adults (80.9%) and adults (79.8%) than by adolescents (75%). Lastly, it became evident that older adults had at least one snack between their daily meals more frequently than adults (88.6% vs. 85.7%) (Table 2).

## Discussion

Eight patterns of daily meal and snack consumption were identified in the analysis of the INA 2017-2018. Generally, the three main meals - breakfast, lunch, and dinner - were part of the daily eating habits of approximately 80% of participants, and the consumption of at least one snack throughout the day was reported by 87% of the Brazilian population. In the present study, it was found that 80% of Brazilians had a meal pattern compatible with the recommendations on meal habits offered by the *Dietary Guidelines for the Brazilian Population* ^2^. However, alternative meal patterns were also observed in the population: about one fifth of participants reported skipping at least one main meal per day, a proportion that almost doubled on atypical days and was more evident among women and adolescents.

In this study, the principle of parsimony ^22^ was followed to choose an objective approach - the EDA ^21^ - to examine meal and snack patterns, which were derived from the combinations of meals and/or snacks according to their consumption by participants in specific days. This helped avoid arbitrariness, eliminating events such as the determination of the number of dimensions proper of the multiple correspondence analysis ^23^ or the number of clusters generated by k-means clustering ^24^. Moreover, patterns generated by correspondence analysis or clustering can be difficult to interpret ^25^. Additionally, Leech et al. ^26^ highlight that the techniques for identifying meal patterns guided by exploratory data analysis have advantages over approaches based on hypotheses that only consider the time of consumption of meals, as they allow for the observation of different eating habits in different segments of the population, including less conventional meal patterns.

A recent narrative review found that the principal component analysis, clustering, latent class analysis, and decision trees were the most commonly used statistical techniques to identify meal patterns in observational studies ^12^. The authors pointed out that meal patterns can be grouped into three categories: (i) temporal patterns, referring to the timing and distribution of meals throughout the day; (ii) content patterns, referring to the combinations of foods within each meal and combinations of meals over the day; and (iii) context patterns, referring to external elements of the meal, such as location, activities done while eating, and the presence or absence of other people. O’Hara & Gibney ^12^ observed that the most common temporal meal patterns were the three meals per day pattern, the breakfast skipping pattern, and a grazing pattern consisting of smaller but more frequent meals. Likewise, in our analysis, temporal meal patterns were identified and the patterns including three main daily meals were found to be the most common.

In the INA 2017-2018, the elevated presence of meal and snack patterns that did not include the three main meals may have resulted from the changes in eating habits owing to the nutrition transition process that had taken place over the last decades ^27^, which ultimately resulted in reduced time for cooking and eating at home and in increases in the consumption of packaged, ready-to-eat products as well as the habit of eating away from home ^28^ ^,^ ^29^. Additionally, meal and snack patterns including one or two main meals were found to be more frequent on atypical food consumption days. Atypical food consumption days are characterized by non-habitual consumption - days like these include weekends, celebrations, festive dates, sick days, and other events causing changes in the daily routine -, while typical food consumption days reflect the individual’s eating routine, i.e., in these days, consumption is closer to usual ^30^ ^,^ ^31^ ^,^ ^32^.

Differences in the definition of eating occasions may affect how meal patterns are characterized and the direction and magnitude of associations with diet quality and health outcomes ^26^ ^,^ ^33^. Leech et al. ^26^ identified the eight most commonly used definitions to describe eating occasions, and the ones employed most frequently considered the time of day and the designation of the respondent or identified participant. Due to the fact that there are multiple ways to define eating occasions, studies on this subject usually detail the criteria used to classify consumption occasions ^34^. In this study, eating occasions were designated by participants when answering a 24hR, and snacks were classified as morning, afternoon, or evening snacks according to the time of day when they were consumed.

Similar to the present study, in the third *National Health and Nutrition Examination Survey*, Kerver et al. ^35^ used participant-identified meal and snack patterns of U.S. adults based on meal and snack/beverage consumption occasions reported in a 24hR. The authors used the five most reported meal and snack combinations to identify the meal patterns, which were divided into the following categories: breakfast + lunch + dinner + two or more snacks (31.6%); breakfast + lunch + dinner + one snack (15.4%); breakfast + dinner + two or more snacks (13.1%); breakfast + lunch + dinner (8.3%); and lunch + dinner + two or more snacks (7.6%). The authors emphasized that meal patterns including the main meals may be associated with diets with better nutritional quality, since individuals who reported the patterns breakfast + lunch + dinner + two or more snacks and breakfast + lunch + dinner + one snack had the highest intakes of all micronutrients examined, except cholesterol, vitamin B_6_, and sodium ^35^.

In the *Santé, Inégalités et Ruptures Sociales* survey (SIRS - *Health, Inequalities and Social Ruptures*), conducted with the adult population of the metropolitan area of Paris (France), Lhuissier et al. ^36^ identified meal patterns by asking three questions: (i) how many times do you eat during the day?; (ii) how many of these eating occasions do you consider as meals?; and (iii) what are your meal times during the day? The authors observed that, similar to the findings of this study, the three-meal pattern was as a strong cultural trait (65.9%) and was more frequent among older individuals (60 years old and over: 77.4%) compared to younger ones (18-29 years old: 54.9%). Furthermore, it appears that the practice of having three main meals a day is more common in Brazil than in Paris. Additionally, while in Brazil men were found to adopt the three-meal pattern more frequently than women, in Paris, the three-meal pattern was more common among women (70.5%) compared to men (60.7%).

In Brazil, meal and snack patterns have been evaluated in studies with adolescents, and most information on meals was obtained using self-administered, close-ended questionnaires about the usual frequency of meals; additionally, meal habits were classified as satisfactory or unsatisfactory depending on whether the main meals (breakfast, lunch, and dinner) were regularly or irregularly consumed ^13^ ^,^ ^15^ ^,^ ^16^ ^,^ ^17^. The present study adds important contributions to the knowledge on this subject in Brazil, as it expands the identification of meal consumption patterns to other age groups, such as adults and older adults. Furthermore, in addition to relying on the respondent’s designation to characterize the meals, this analysis used an exploratory statistical approach to identify the possible daily combinations of meals and snacks and recognize the meal patterns that represent the eating occasions in Brazilians’ routines.

The possible limitations of this study are related to the fact that the 24hR was used to obtain information on meal and snack habits. The 24hR allows researchers to obtain detailed information on food consumption, including the composition and time of consumption of meals and snacks ^26^ ^,^ ^35^, but it relies on the respondents’ memory and may be subject to underreporting. In the INA 2017-2018, strategies to reduce misreport included following the Automated Multiple-Pass Method ^19^ to apply the 24hR with the support of computational resources. Moreover, as the 24hR is not associated with systematic errors, it is often used in extensive population-based surveys carried out worldwide ^37^. Furthermore, each participant informed whether the reported food consumption corresponded to a typical or an atypical food consumption day. Lastly, the analyses showed that there were no significant differences between the meal and snack patterns identified in the first and the second 24hR.

One strength of this study is its large, nationally representative sample, which included Brazilians aged 10 years and over and provided robust estimates. In addition, the eating occasions were identified objectively, based on participants’ designation and the time of consumption. Another strong point of this study is the fact that it took a holistic approach to meal and snack habits rather than focusing on isolated meals, which made it possible to assess participants’ compliance with dietary recommendations and to identify alternative meal and snack patterns. This approach allows future studies to explore the association between dietary habits and health outcomes, an aspect that has been highlighted by chrono-nutrition studies ^38^.

Traditionally, nutritional epidemiology research has focused on the assessment of dietary intake and patterns; however, the focus on dietary habits is still restricted ^26^, especially in low- and middle-income countries such as Brazil. As pointed out by Popkin & Ng ^39^, the process of nutritional transition, which takes place more rapidly in middle and low-income countries than in developed ones, includes changes in eating habits, for example, an increase in the number of eating occasions and in the contribution of ultra-processed products to total energy intake. Various studies have indicated that the frequency, location, and nutritional composition of main meals and snacks are associated with diet quality and health ^12^. Moreover, specific meal patterns may be associated with better diet quality and nutrient intake, considering that these patterns may reflect healthier lifestyle choices ^35^. In Brazil, Rodrigues et al. ^16^ carried out a cross-sectional school-based study to evaluate the quality of adolescents’ diets and observed that the habit of skipping meals was associated with diets of low nutritional quality, especially those with low consumption of fruits and vegetables and high consumption of solid fats, added sugar, and alcoholic beverages. Therefore, acknowledging meal consumption patterns is central for targeting and tailoring actions to promote healthy eating.

These findings may be an initial step towards the recognition of meal and snack habits in Brazil. Further studies should assess the socioeconomic and demographic characteristics associated with each meal and snack pattern, as well as the distribution of energy intake and other dietary features specific to these patterns.

## Conclusion

Eight meal and snack patterns were identified among Brazilians aged over 10 years. It is noteworthy that a significant portion of the population studied consumed three main meals a day, while less than 20% did not report consuming snacks. Skipping at least one of the main meals on atypical food consumption days was almost twice as frequent as on typical days. Women and adolescents skipped at least one of the main meals to a greater extent than men, adults, and older adults. By evaluating a nationally representative sample, this study contributes to a better understanding of Brazilians’ eating habits.
